# Absolute Quantification of Selected Proteins in the Human Osteoarthritic Secretome

**DOI:** 10.3390/ijms141020658

**Published:** 2013-10-15

**Authors:** Mandy J. Peffers, Robert J. Beynon, Peter D. Clegg

**Affiliations:** 1 Department of Musculoskeletal Biology, Institute of Ageing and Chronic Disease, University of Liverpool, Leahurst, Chester High Road, Neston, Cheshire, CH64 7TE, UK; E-Mail: pclegg@liv.ac.uk; 2 Protein Function Group, Institute of Integrative Biology, University of Liverpool, Biosciences Building, Crown Street, Liverpool, L69 7ZB, UK; E-Mail: rbeynon@liv.ac.uk

**Keywords:** cartilage, human, osteoarthritis, QconCAT, secretome

## Abstract

Osteoarthritis (OA) is characterized by a loss of extracellular matrix which is driven by catabolic cytokines. Proteomic analysis of the OA cartilage secretome enables the global study of secreted proteins. These are an important class of molecules with roles in numerous pathological mechanisms. Although cartilage studies have identified profiles of secreted proteins, quantitative proteomics techniques have been implemented that would enable further biological questions to be addressed. To overcome this limitation, we used the secretome from human OA cartilage explants stimulated with IL-1β and compared proteins released into the media using a label-free LC-MS/MS-based strategy. We employed QconCAT technology to quantify specific proteins using selected reaction monitoring. A total of 252 proteins were identified, nine were differentially expressed by IL-1 β stimulation. Selected protein candidates were quantified in absolute amounts using QconCAT. These findings confirmed a significant reduction in TIMP-1 in the secretome following IL-1β stimulation. Label-free and QconCAT analysis produced equivocal results indicating no effect of cytokine stimulation on aggrecan, cartilage oligomeric matrix protein, fibromodulin, matrix metalloproteinases 1 and 3 or plasminogen release. This study enabled comparative protein profiling and absolute quantification of proteins involved in molecular pathways pertinent to understanding the pathogenesis of OA.

## 1. Introduction

Articular cartilage, an avascular connective tissue, provides a nearly frictionless bearing surface for transmitting and distributing mechanical loads between the bones of the skeleton [1]. The chondrocyte, the sole cell type [2] is embedded within an extracellular matrix (ECM) whose unique load bearing properties are dependent upon its structural composition and organisation particularly the interactions between collagens and proteoglycans [3]. Progressive articular cartilage loss leads to joint pain and dysfunction that is clinically identified as osteoarthritis (OA). In OA the normal equilibrium between matrix deposition and degradation is disrupted resulting in progressive loss of important ECM components, especially aggrecan and collagens.

Mass spectrometry (MS) has emerged as an important analytical tool for protein analysis with MS-based proteomics enabling proteins within a sample to be identified and quantified. Cartilage proteomic studies have permitted the investigation of cartilage proteins in both the intact cartilage tissue [4,5] and the cartilage secretome [6–9] with a number of studies reporting IL-1 driven protein secretion from cartilage ECM [10,11]. The cartilage secretome is defined as the proteins identified in the media surrounding the chondrocyte or explants and includes proteins secreted or shed from the cell surface, plus intracellular proteins released into the supernatant due to cell lysis, apoptosis or necrosis [12]. In cartilage explant studies, proteins released into media by chondrocytes and ECM may be similar to proteins released *in vivo* in cartilage degradation [4]. Data from these studies has enabled improved understanding of OA pathogenesis [13]. An accepted method of studying matrix metabolism in experimental investigations of OA *in vitro* is alteration of the secretome by addition of pro-inflammatory cytokines to explants [14,15]. Indeed, cytokine stimulation of normal and OA cartilage explants has been used in numerous studies to initiate a catabolic response [16–18] and assess different facets of the degradative process.

The two types of protein quantification are absolute quantification, which determines real amounts of a protein in terms of concentrations, for example, as copies per cell, and normally uses external or internal standards and relative quantification. The latter determines differences in protein abundance relative to an internal control but does not report on absolute concentrations. Within cartilage research there is a need for absolute quantitative MS in order to define proteins in tangible amounts. This will aid the understanding of and define how protein content of chondrocyte ECM alters in both ageing and disease. Moreover, such data will provide necessary information for mathematical modelling of biological systems. Although relative quantification of the cartilage secretome has been undertaken in numerous studies [8,19–21], these experiments focus on “discovery” proteomics and the detection of differentially expressed proteins. Few studies have attempted to quantify the cartilage secretome in exact amounts. Whilst this work has enabling biomarker discovery to progress [6,20], a more detailed knowledge of the quantities, interactions and dynamics of matrix components and the protease enzymes involved in degradation will increase our understanding of the as yet undefined mechanisms involved in ECM destruction typical of OA. For example, knowledge of the exact nature of protease/tissue inhibitors of metalloproteinase (TIMP) will further our comprehension of OA pathogenesis, which in turn could aid in the discovery of treatments. Metabolic isotope labelling in culture using stable isotope labelling of amino acids in cell culture (SILAC) has been employed in comparative cartilage studies [22]. Whilst SILAC is suitable for the quantification of the same protein under different conditions, it is not suitable for quantification of different proteins under any conditions. Furthermore, such data is dimensionless leading to difficulties in interpretation.

The two current approaches to absolute quantification are label-free and label-mediated quantification. Label-free methods are based on the direct measurement of the MS acquired signal. When constituent peptides are produced following protein digestion and are converted into ions, the most abundant proteins will produce the most ions and thus the greatest signal intensities [23]. This method provides acceptable quantification for the high abundance components of a sample but suffers at the low abundance range due to technical variance [24]. Stable isotope labelled quantification includes the use of chemically synthesized peptide standards known as AQUA peptides [25] and QconCAT [26]. QconCAT are artificial proteins that permit highly accurate parallel absolute quantification of large sets of analyte proteins [26]. These constructs are a set of mass-tagged internal standard peptides (each internal standard of the stable-isotope labeled reference peptide is known as a “Q-peptide”) with sequences unique to the proteins of interest. Multiple peptides are concatenated into a synthetic gene and expressed as a heterologous QconCAT protein in bacterial cultures [27,28], allowing large numbers of biological samples to be analyzed in a cost effective and reliable manner [29]. The proteins selected in this QconCAT included ECM proteins and proteases relevant to OA pathology that were of interest to our group. For example, the role of the membrane-bound protease MMP-16 in cartilage degradation is controversial [30] and of interest to us. We wished to use our QconCAT as a tool to quantify proteins in a number of different projects as it enables the absolute quantification of many proteins in a single experiment. Whilst there are commercially available enzyme linked immunosorbent assays (ELISA) to some of these proteins such as MMP-13, for others, such as link proteins, these are not available. Furthermore, QconCAT quantification has the advantages of sensitivity, specificity and the ability to quantify a wide dynamic range [31]. Both AQUA and QconCAT rely on the MS quantification of a known amount of isotope-labelled peptide standard relative to the otherwise identical non-labelled within an analyte peptide. This approach benefits from a high level of sensitivity with quantification possible to the atomole level. A number of papers have used QconCAT technology to quantify multiple proteins including muscle development proteins [27], glycolytic proteins in yeast [32], surface proteins in *Schistosoma mansoni* blood fluke [33], host response to bovine mastitis pathogens [34] and cohesion interactions in human cell lines [35].

The aim of this study was to develop and test a targeted quantification method for the multiplexed analysis of proteins involved in the pathogenesis of OA. To achieve this we have used label-free data to give a preliminary assessment of the OA secretome protein profile and protein abundance. We then used absolute quantification to validate and give absolute baseline values for a number of key matrix proteins. We hypothesize that there are measurable changes in protein abundance in the human OA secretome following cytokine stimulation that can be absolutely quantified using QconCAT.

## 2. Results

### 2.1. Comparative Analysis by Mass Spectrometry

**Figure 1:**
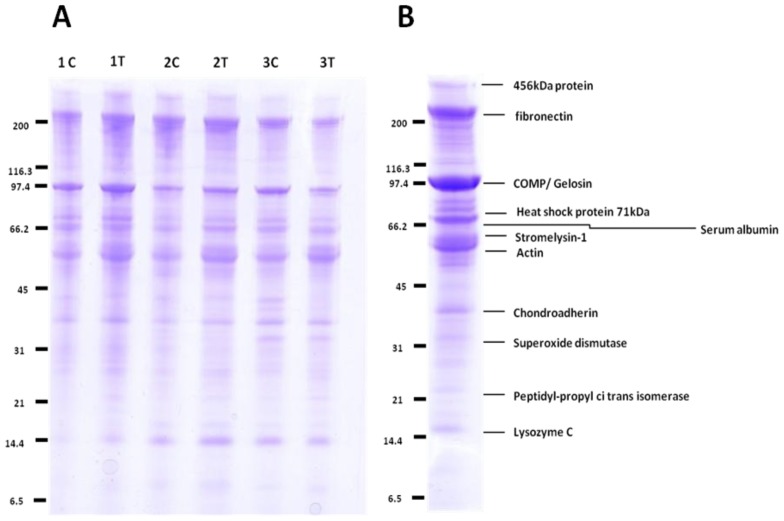
1D-SDS-PAGE of the cartilage OA secretomes demonstrated little difference in the profiles following IL-1β treatment. (**A**) OA human articular cartilage explants (*n =* 3) were cultured in media supplemented with 10 ng/mL IL-1β (T) or un-supplemented media (C). Culture media were collected at two days for further analysis by SDS-PAGE and staining with Coomassie Brilliant Blue. Equal protein loading of 20 μg of protein per well allowed a qualitative comparison of the secretomes; (**B**) the most abundant proteins in the media marked at the positions of the bands were excised from the gel, trypsin digested, and the protein content of each single band was analysed using peptides identified using LC-MS/MS. Proteins indicated on the gel correlate to the size and are the primary protein identified in the corresponding gel analysis.

All cartilage was graded as severe OA using a modified Mankin scoring [36] (original score proposed by Mankin *et al.* 1970) [37] with scores of between 9 and 13 out of 14. 1D-SDS-PAGE of the cartilage OA secretomes (Figure 1A) demonstrated no significant difference in the profiles of the control and IL-1β treatment using densitometry (data not shown).

The repertoire of proteins secreted by explants was then analysed by MS (Figure 1B). Proteins included in the results had a Mascot score >40 with two or more identifying peptides and a confidence interval of 95%. The cartilage explant secretomes contained a number of cartilage matrix proteins as expected, plus proteins associated with catabolic aspects of cartilage matrix turnover such as MMP-3.

Explant experiments were undertaken in duplicate and in-solution tryptic digestion of media was then performed in duplicate. This gave a total of four secretome replicates per donor per condition. We processed these samples for mass spectrometry as described and performed quantitative analysis with Progenesis™ LC-MS software. All identified features in the 2-D maps were aligned between samples, normalised and assigned to control or IL-1β treatment groups. ANOVA was performed on normalised peptide intensities and Mascot was then used to identify all features with MS/MS data against the UNIHUMAN database with search results implemented into the experiment file. Peptide identifications were merged into non-redundant protein identifications.

### 2.2. Label-Free Protein Profiling of the OA Secretome

A total of 278 proteins were identified including aggrecan, fibromodulin, cartilage oligomeric matrix protein, fibronectin, matrix metalloproteinase 1 and 3, link protein and plasminogen (Table S1). Gene ontology determined that 53% of genes were identified as “secreted” and 18% were identified as belonging to ECM. Of these, 242 were identified with ≥2 unique peptides.

Nine proteins showed greater than two-fold differential expression between control and IL-1β stimulation groups (*p <* 0.05) in expression (Table 1). Four of these were increased following IL-1β stimulation and five were decreased.

In order to both quantify proteins of interest and validate findings of some of the secreted proteins in our study, we employed QconCAT technology.

### 2.3. QconCAT Protein Design, Expression and Validation

The QconCAT containing proteins of interest to our research studies was designed with two peptides per protein. The QconCAT had an average mass of 58.8 kDa which included the *N*-terminal fibrinopeptide and *C*-terminal glufibrinopeptide (to allow quantification of the QconCAT) together with a hexahistidine tag for purification. The proteins were selected with their respective accession numbers, peptide sequences and *m*/*z* values of the protonated molecules, and are presented in Table 3. Following gene synthesis into pET21a vector and successful transformation into *E. coli*, induction of expression and purification led to a recombinant protein band which migrated on SDS-PAGE with mobility consistent with an approximate mass of 58 kDa, indicating correct expression of the QconCAT.

The putative QconCAT protein band excised from the gel following purification was trypsin digested and analysed with MALDI-TOF mass spectrometry. In total, 27 out of 40 predicted peptides from the product ion spectra were identified (Figure S1). Although the peptides were equimolar in proportions within the QconCAT, the MALDI spectra demonstrated the expected variability of ionisation between individual peptides. Furthermore, the peptides glufibrinopeptide and fibrinopeptide were identified, indicating full length expression of the QconCAT, as these peptides are positioned at the beginning and end of the sequence cassette. In order to further validate the QconCAT we identified Q-peptides using LC-MS/MS on the LTQ-Orbitrap Velos from an in-solution tryptic digestion, with 100 fmol on column QconCAT. MASCOT identified 91% sequence coverage of the QconCAT (data not shown). DGFFYFFHGTR and VARPAQLASPTR demonstrated weak fragmentation patterns during the identification and assembly of peptide transitions lists for SRM experiments. No miscleavages were identified indicating complete QconCAT proteolysis.

A 200 mL bacterial culture, grown to a cell density of OD*_A_*_600_ 0.6–0.8, yielded 33 μg of the QconCAT. The identity and chromatographic retention time of the Q-peptides from the unlabelled and labelled QconCAT recombinant proteins were established by preliminary tandem MS analyses of pure QconCATs. QconCAT peptides were labelled to 98.7%, reflecting the quality of the starting isotopes [^13^C_6_]Arg and [^13^C_6_]Lys.

The linearity of the response for the QconCAT peptides was established prior to these analyses using labelled and unlabelled QconCAT mixed at different ratios using 50 fmol QconCAT loaded on column in a SRM experiment using the XEVO TQ (Figure S2).

### 2.4. Peptide Choice and Detectability

We chose two peptides per protein in an attempt to circumvent possible problems such as poor peptide ionisation or detectability by the mass spectrometer which would prevent quantification. For proteins we wished to quantify in the cartilage secretome with a SRM experiment using the QconCAT, a simple classification was applied to peptides as described by Brownridge *et al*. 2011 [38]. Peptides were classified for quantification purposes as A, B, C for a particular protein loading. “Type A” quantifications were defined as were both the QconCAT and native peptides observed. For “Type B” quantifications, the peptide was detected in the QconCAT but not in the native peptide, and for these peptides, sample protein abundance sets the limit on detection. Neither QconCAT nor native peptides are detected in “Type C” quantification, typically due to poor peptide fragmentation or chromatographic behaviour (Table 2). Of the 40 peptides composite of the QconCAT, we wished to use 30 peptides in order to quantify their constituent proteins within the cartilage secretome. Of these, 12 were “Type A”, 10 were “Type B”, and eight were “Type C”. This enabled the quantification of seven proteins in our human OA secretome using the criterion that at least one Q-peptide per protein was detected in all samples.

### 2.5. Quantification of Proteins Using SRM

SRM of multiple product ions were used for quantification as it provided a sensitive method for targeted analyte identification and quantitation. When possible, two transitions per peptide were used for quantification. Transitions were defined using Skyline software [39] and selected after monitoring for the greatest intensity fragments using 50 fmol QconCAT digest on the XEVO TQ. Fragmented y-ions were selected in order to differentiate labelled and unlabelled peptide as the *C*-terminal residue contained the isotope-labelled amino acid. In addition, to maximise specificity we selected transitions whose *m*/*z* was greater than the parent ion *m*/*z* (Table S2). We were unable to identify any adequate transitions for quantification purposes for two peptides—VGPVSVAIDASLTSFQFYSK (cathepsin K) and DGFFYFFHGTR (MMP-1)—emphasizing the need to include at least two peptides within a QconCAT to quantify a protein.

**Figure 2:**
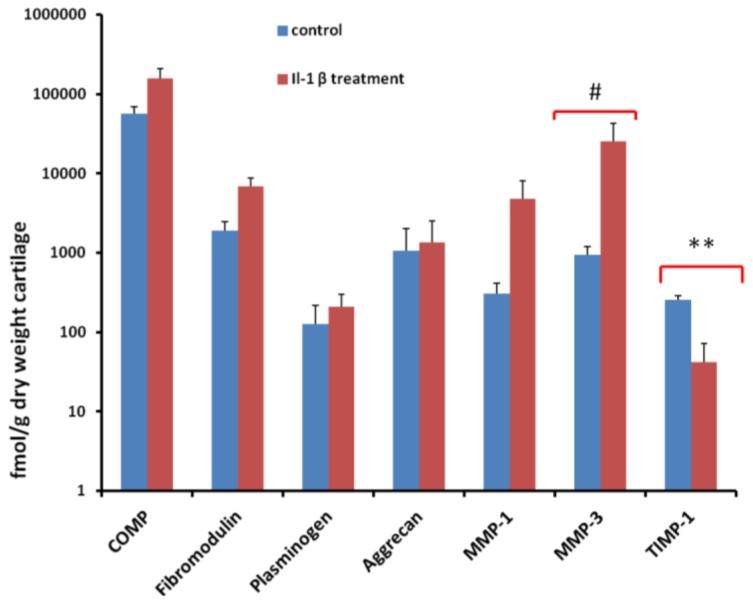
Proteins measured in human secretome media using QconCAT. Extracted ion chromatograms were performed for each peptide and the total ion count used to determine the ratio of light peak area/heavy peak area at a given QconCAT loading. The protein abundance in the media was then calculated based on the amount of total protein in the media sample. This was then normalised to the dry weight of explants. Mean concentrations and ±SEM (*n =* 3) are indicated. Data were evaluated using mixed effect linear regression. ** indicates significant difference relative to control at the *p <* 0.01 level; # indicates *p =* 0.06.

The seven proteins quantified using an SRM approach were aggrecan, COMP, fibromodulin, MMP-1, MMP-3, plasminogen and TIMP-1 (Figure 2). For COMP we were able to quantify using both peptides: SSTGPGEQLR and DTDLDGFPDEK. DTDLDGFPDEK consistently produced a lower ratio of light peak area/heavy peak area compared to SSTGPGEQLR. Analysis of MASCOT data of analyte digests revealed that occasional miscleavage of DTDLDGFPDEK was evident resulting in an underestimation of protein abundance when this peptide was used for absolute quantification. We therefore used SSTGPGEQLR for quantification of COMP. Following IL-1β treatment, there was an apparent increase in aggrecan, COMP, fibromodulin, MMP-1, MMP-3 and plasminogen and a reduction in TIMP-1. The only protein that achieved statistical significance was TIMP-1, with a reduction in IL-1β treated samples (*p =* 0.0017), although MMP-3 exhibited a trend (*p =* 0.06).

### 2.6. Validation of SRM Results for MMP-3 Using Immunoblotting

**Figure 3:**
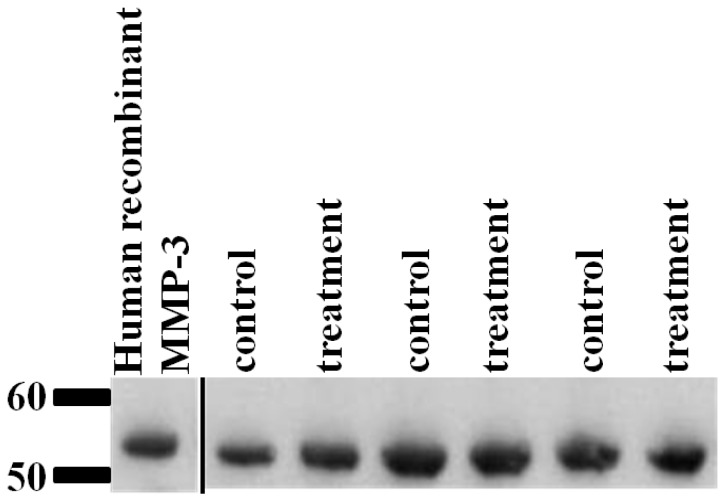
Cartilage explants treated with Il-1β did not demonstrate altered MMP-3 protein expression. Western blot analysis using antibodies to MMP-3 on cartilage explant supernatant cultured with and without IL-1β. Images of western blots for control and IL-1β treated (treatment) cartilage explants. Volumes of supernatant loaded were normalised to dry weight of cartilage. Human recombinant MMP-3 was used as a positive control. There was no difference in the relative intensity the bands following analysis in ImageJ (data not shown).

Quantitative immunoblotting confirmed there was no significant difference in the abundance of MMP-3 in cartilage explant supernatant following IL-1β treatment (Figure 3).

## 3. Discussion

Cartilage proteomics is developing from simple protein identification through to quantitation. For OA research, absolute quantitative proteomics will enable further biological questions to be addressed, by facilitating the experimental determination of absolute protein amounts. However, there have been few studies able to absolutely quantify cartilage secreted proteins and experiments have employed relative quantification approaches using platforms including 2D gel approaches [9,21], SILAC studies [40], isobaric tags for relative and absolute quantitation (iTRAQ) [8], and quantitative western blotting [41]. Absolute quantification techniques and its variations have been utilized to measure absolute amounts of a given peptide, allowing quantitative comparisons of different proteins. QconCAT technology allows the cost efficient production of heavy isotope labelled standards. The aim of devising this human cartilage QconCAT was to provide an accurate, low-cost method able to support large scale protein quantification in cartilage studies.

The established cartilage explant model mimics the catabolic events that occur in OA [18,42]. For the purposes of this study, cartilage from osteoarthritic joints was used since we wished to evaluate quantitative proteomic techniques including QconCAT in cartilage research. It would have been advantageous to compare the results to normal cartilage; though, direct comparison may be obscured due to inherent heterogeneity of normal and OA cartilage and difficulty in recruiting age-matched controls. However, there is precedence in using such samples as pathological human cartilage has been utilized to examine the role of cytokines and the targeted analysis of protein expression alterations in OA [43]. Cartilage explant cultures enable chondrocytes to be retained within their ECM and keep their phenotypic stability. The ECM also provides native substrates for proteolysis and protein release.

Using 1D-SDS-PAGE, we were able to take a qualitative proteomics approach in order to identify the predominate proteins in the HAC OA secretome. Densitometry of the gel did not identify differential protein expression following 48 h stimulation with IL-1β and this may be due to insufficient exposure time to the cytokine or previous findings by others that responsiveness to IL-1β is reduced in late stage OA chondrocytes [44]. Furthermore, results from the label-free study indicated very few differentially altered proteins in our model (nine out of 278 proteins identified) in comparison to our other studies in normal cartilage stimulated with IL-1β, which demonstrated over 100 differentially expressed proteins [45].

High-throughput proteomic technologies created large data sets posing challenges in interpretation. Therefore, a proteomics tool called Progenesis LC-MS™ was utilized, enabling the analysis and relative quantification of proteins in our experiment. Following Progenesis LC-MS™ analysis, we applied insightful data mining using the bioinformatic tool DAVID [46] in order to interpret the data in relationship to protein location and function. As predicted, a large proportion (53%) of proteins identified by GO, which uses statistical analysis to validate results, were secreted. Whilst the dataset of differentially expressed proteins identified by Progenesis™ was small, it was interesting that a number of them were involved in inflammation and the innate immune response. Inflammation plays an important role in the pathogenesis of OA [47], and following proinflammatory cytokine stimulation of already diseased cartilage, it was not surprising that proteins, implicated in pathways associated with rheumatoid arthritis and OA, were identified. It is hypothesised that inflammation might actually be driven by the fragments such as fibronectin [48] that are released by cartilage degradation, through activation of the innate immune responses. More recently it has been identified that inflammatory complement cascade has a key role in the pathogenesis of OA [49]. One interesting finding was the reduction in decay accelerating factor splice variant (DAF) in IL-1β stimulated explant media. DAF belongs to the complement system and protects cells from complement mediated lysis. Immunohistochemistry studies identified an increase in OA cartilage compared to normal [50] and there was an increase in transcript in macroscopically affected OA joint cartilage compared to intact cartilage in the same joint [51]. It is possible that aberrant regulation of DAF is occurring due to IL-1β stimulation of already diseased cartilage. Further work into the role of this protein is warranted. Finally, as inflammation is an early and persistent event, the involvement of joint tissues in OA could be monitored by quantifying levels of a panel of markers such as the inflammatory factors identified as differentially expressed in this study: DAF, growth-regulated alpha protein and IG alpha-1 chain C region.

Many ECM proteins and proteases were identified in the secretome using LC-MS/MS. However, some interesting proteins, particularly proteases/protease inhibitors, were not identified. We hypothesised that a more targeted SRM approach that increases sensitivity [52] would enable the identification and subsequent quantification of further proteins. Therefore, a human cartilage QconCAT was designed. This approach identified and quantified aggrecan, COMP, fibromodulin, link protein, MMP-1, MMP-3 and TIMP-1 from within the secretome.

A strategy was used where two peptides were included per protein in the QconCAT to allow quantification with at least one peptide should one fail. For the eight proteins identified in MS/MS data and then quantified with SRM, we were reduced to a single peptide for quantification for all peptides except COMP. For this protein, a single peptide was nominated for quantification purposes. The main reason for redundant Q-peptides was poor peptide selection, resulting in “Type C” peptides within our QconCAT. The primary reason for “Type C” peptides was poor fragmentation of the parent peptide resulting in inadequate transitions for quantification using an SRM approach. Interestingly both link protein peptides were identified in the label-free experiments (where both MS and MS/MS are used in protein identification), but we were unable to quantify either peptide. One Q-peptide, FYYLIHPTK, was identified as a “Type C” peptide. The other peptide, GGSDSDASLVITDLTLEDYGR, was readily detected down to 0.1 fmol in the standard but was not detected in analyte. It is possible that there may be a previously unidentified PTM on this peptide. In addition, this peptide displayed a poor fragmentation pattern as demonstrated by the identification of only a single transition for SRM experiments.

For aggrecan, EVVLLVATEGR was used in quantification experiments, as the other peptide, LEGEVFFATR, was not detected in any sample. The tryptic cleavage site at the *N*-terminal of this peptide was close to an aspartic acid, possibly leading to miscleavages, although no evidence was found for this in MS/MS data. While we were able to quantify COMP using both peptides, DTDLDGFPDEK consistently produced a lower ratio of light peak area/heavy peak area, possibly resulting from miscleavage due to the positioning of an aspartic acid residue adjacent to the cleavage site in the analyte. Thus, SSTGPGEQLR was utilized in the quantification of COMP. For the proteins fibromodulin, MMP-1, TIMP-1 and plasminogen, one peptide per protein was a “Type C” peptide, resulting in a single peptide being used for quantification by SRM.

Il-1β is one of the most significant cytokines in OA [53], and is assumed to cause damage to OA cartilage through both the induction of protease expression, resulting in cartilage matrix degradation [14,54] and reduction in the expression of anabolic genes such as aggrecan and COL2A1[55]. This results in the anabolic-catabolic discrepancy characteristic of OA. Here, we examined OA cartilage degeneration in culture using secretome protein identification and both relative and absolute quantification. Relative quantification identified the induction of MMP-3 protein expression following IL-1β stimulation. The absolute quantification also revealed an increase in MMP-3 in all donors in each replicate, although this did not reach statistical significance (*p <* 0.06). Whilst others identified an induction of MMP protein expression along with degradation of matrix constituents in OA HAC explants [56], the induction of MMP-3 demonstrated in this study is not accompanied by an increase in ECM degradation. This could be due to the short time scale of the experiment or that there is a reduced responsiveness to IL-1β in late stage OA chondrocytes.

One of the most abundant proteins quantified here was COMP, a non-collagenous matrix protein. Its presence in the secretome corresponded to previous studies of cartilage explants [57]. COMP organizes ECM assembly [58] and attaches the chondrocyte to the ECM [59]. Considered as a marker of cartilage breakdown, it has been studied as a biological marker [60,61]. Measurements of intact COMP and fragments thereof in synovial fluid or serum have been shown to correlate to cartilage destruction in OA patient studies [62] and so it is no surprise that it is abundant in the secretome.

Fibromodulin, a collagen-binding protein [63], was also quantified. This protein protects the surface of collagen type I and II fibrils from proteolysis by MMPs [64]. Cleavage products of fibromodulin have been identified during IL-1 stimulation of cartilage explant studies *in vitro* [63,65] and, as such, cleavage of fibromodulin may represent an important initial episode that interrupts the collagen fibrillar network leading to more sites for proteases to cleave collagen further. Furthermore, it has been suggested that some ECM proteins including fibromodulin become endogenous catabolic factors during joint damage and stimulate innate immune pathways via complement activation [66]. Thus, the presence of fibromodulin in relative abundance within the OA secretome presents a means by which ongoing joint damage may be further precipitated.

Plasminogen, a serine protease and important activator of pro-MMPs, has been demonstrated to induce cartilage degradation [67]. Its production is stimulated by Il-1β in cartilage [68]. Although it was not evident in initial MS/MS experiments, it was quantified using SRM. SRM experiments are unique in their ability for reliable quantification of analyte of low abundance in complex mixtures [69]. This demonstrates the advantage of our QconCAT approach to proteomics experiments in the study of low abundance proteins such as plasminogen involved in OA.

MMP activity is regulated by a family of tissue-specific inhibitors including TIMP-1 [70]. Presently, it is believed that the local balance of MMPs and TIMP activities is crucial for cartilage homeostasis. TIMP concentrations generally far exceed the concentration of MMPs in tissue and extracellular fluids, thereby limiting their proteolytic activity to focal pericellular sites by binding to the MMP active sites [71]. TIMPs also inhibit cleavage of proteoglycans by aggrecanases [72]. In the IL-1β stimulated secretome, MMP-3 concentrations exceeded TIMP-1 concentrations as identified using relative and absolute quantification. Interestingly, in the QconCAT study, TIMP-1 was the only protein significantly affected by cytokine stimulation agreeing with the label-free study. TIMP-1 mRNA has been previously identified in OA [73] and rheumatoid arthritis [74]. Our results agree with other studies demonstrating that IL-1β stimulation has a marked inhibitory effect on TIMP-1 expression by chondrocytes [75].

Bringing the results of the different quantification methodologies together it would seem that there is good agreement with the findings from the two studies when proteins were identified as significantly differentially expressed in the secretome. MMP-3 was increased significantly in the label-free study and increased to near significance in the QconCAT study. TIMP-1 expression was identified as significantly reduced using both methodologies. The ECM proteins aggrecan, fibromodulin and COMP were not differentially expressed in the study using either method.

## 4. Experimental Section

### 4.1. Peptide Selection, Preparation and Purification of QconCAT

We selected 20 proteins of interest in cartilage degradation. Two proteotypic tryptic peptides per protein were selected from the Global Proteome Machine database (www.thegpm.org) and the Human PeptideAtlas (www.peptideatlas.org) based on published criteria [28] including their suitability score, physicochemical properties deemed to promote MS detectability, and uniqueness to a given protein. In addition, amino acids that were prone to oxidation and miscleavage were avoided and all peptides terminated with either lysine or arginine (Table 3). Peptides were arranged in sequence context where possible. In the native protein, the amino acid sequence prior to the Q-peptide was noted and the peptides in the QconCAT ordered where possible to mimic this in order to optimise digestibility.

The transformation, expression and purification of QconCAT has been previously described in detail [27]. Briefly, following synthesis of the gene by PolyQuant GmBH (Entelechon, Germany), the QconCAT was ligated into the expression vector pET-21a and expressed in *Escherichia coli* cultured in minimal media (1 × M9 salts, 1 mM MgSO_4_, 0.1 mM CaCl_2_, 0.00005% (*w*/*v*) thiamine, 0.2% (*w*/*v*) glucose, unlabeled amino acids at 0.1 mg/mL or 0.2 mg/mL histidine, tyrosine, phenylalanine, proline and tryptophan all Sigma-Aldrich (Gillingham, Dorset, UK)) supplemented with ^13^C_6_ analogues of arginine and lysine containing stable isotope labelled amino acids. Once cells achieved mid log phase (OD_660nm_ 0.6–0.8) expression was induced by addition of 1 mM IPTG Isopropyl β-D-1-thiogalactopyranoside (Sigma-Aldrich, Gillingham, Dorset, UK). After 5 h of induction, cells were harvested by centrifugation at 1400 × *g* at 4 °C for 15 min. Cell lysis was undertaken using BugBuster Protein Extraction Reagent (Merck Chemicals, Nottingham, UK).

Inclusion bodies were first re-dissolved in 20 mM phosphate buffer, 6 M guanidine chloride, 0.5 M NaCl, 20 mM imidazole, pH 7.4. They were then solubilised using sonication, followed by purification using immobilised metal affinity columns; Ni-MAC (Novagen, Darmstadt, Germany). The purified QconCAT protein was desalted three times by dialysing against 100 volumes 10 mM ammonium bicarbonate, pH 8.5, 1 mM dithiothreitol (DTT) for 2 h changing the buffer each time.

### 4.2. Characterisation of QconCAT

The homogeneity of the QconCAT was determined by the in-gel digestion of a protein band corresponding to the expected molecular mass for the QconCAT of 58 kDa. Briefly, a 5 μg aliquot of purified QconCAT protein was separated on a 12% SDS-PAGE gel (50 min, 200 V), fixed with 40% methanol and 10% acetic acid then stained with Coomassie blue. In-gel digestion was undertaken as previously described [76]. 1 μL of the digest was mixed with 1 μL of α-cyano-4-hydroxycinamic acid (CHCA; Sigma, Poole, UK) in 50% (*v*/*v*) acetonitrile (ACN)/0.1% (*v*/*v*) trifluoroacetic acid (TFA) and 1 μL spotted on a MALDI plate. Positive-ion MALDI mass spectra (MS) were obtained using an Ultraflex (Bruker, Bremen, Germany) in reflector mode over *m*/*z* range 900–4500. Monoisotopic masses were collected from centroids of raw unsmoothed data.

### 4.3. Cartilage Isolation and Explant Culture

Human articular cartilage (HAC) was obtained following total knee arthroplasty due to OA with informed consent and ethical approval. Full thickness cartilage that appeared macroscopically intact and normal was harvested from the entire surfaces of three male donors aged between 69 and 84 years.

Cartilage was diced into explants of approximately 2 mm, mixed and placed in complete medium Dulbecco’s modified Eagle’s medium (DMEM), supplemented with foetal calf serum (10% *v*/*v*), 100 U/mL penicillin, 100 U/mL streptomycin (Invitrogen, Paisley, UK) 500 ng/mL amphotericin B (BioWhittaker, Lonza, San Diego, California, USA). Explants were washed twice with serum-free DMEM (to deplete serum and synovial proteins) and allowed to equilibrate in complete medium for 24 h at 37 °C in 5% CO_2_ in 12 well plates (2 mL/well). Media was then replaced with serum-free DMEM prior to incubation, supplemented with or without human recombinant IL-1β (10 ng/mL; R & D Systems) in dimethyl sulfoxide (DMSO) diluent. After 48 h, media was removed, centrifuged to remove debris and protease inhibitors (Complete Protease Inhibitors, EDTA-free, Roche, Lewes, UK) added. Samples were stored at −80 °C prior to downstream analysis. Protein concentrations of supernatants were estimated by Bradford assay (Thermo Scientific, Rockford, IL, USA). Cartilage explants were lyophilized to obtain a dry weight for normalisation.

### 4.4. 1-D SDS PAGE Separation and In-Gel Trypsin Digestion

Cartilage extract secretomes of control and treatment condition for all donors were analyzed by one dimensional sodium dodecyl sulphate polyacrylamide gel electrophoresis (1D-SDS-PAGE) to assess quantitative/qualitative differences in protein profiles loading 20 μg of protein into each lane. In-gel tryptic digests of bands of interest from the 1D-SDS-PAGE was undertaken as previously described [76]. ImageJ software (http://rsbweb.nih.gov/ij/) was used to quantify bands using densitometry.

### 4.5. Protein Identification of In-Gel Digests by Linear Ion Trap Quadruple (LTQ) Velos Mass Spectrometry

Digested samples were analysed by LC-MS/MS using an UltiMate^®^ 3000 Rapid Separation LC (RSLC, Dionex Corporation, Sunnyvale, CA, USA) coupled to a LTQ Velos Pro (Thermo Fisher Scientific, Waltham, MA, USA) mass spectrometer. Peptides were concentrated on a pre-column (20 mm × 180 μm internal diameter (ID), Waters, Manchester, UK). The peptides were then separated using a gradient from 99% A (0.1% formic acid (FA) in water) and 1% B (0.1% FA in ACN) to 25% B, in 45 min at 200 nL min^−1^, using a 75 mm × 250 μm ID 1.7 μM BEH C18, analytical column (Waters, Manchester, UK). Peptides were selected for fragmentation automatically by data dependant analysis.

Raw spectra were converted to mascot generated files (mgf) using Proteome Discoverer software (Thermo, Hemel Hempstead, UK). The resulting mgf files were searched against the Human IPI database sequence databases using an in-house Mascot [12] server (Matrix Science, London, UK). Search parameters used were: peptide mass tolerances, 10 ppm; fragment mass tolerance, 0.6 Da, 1+, 2+ and 3+ ions; missed cleavages, 1; instrument type, ESI-TRAP. Modifications included were: fixed; carbamidomethyl cysteine and variable; oxidation of methionine. Data produced were searched using Mascot (Matrix Science, London, UK), against the Human IPI database with taxonomy of Homo sapiens selected. Data were validated using Scaffold (Proteome Software, Portland, OR, USA).

### 4.6. In-Solution Tryptic Digestion and Mass Spectrometry Using Linear Ion-Trap Orbitrap Mass Spectrometer (LTQ-Orbitrap Velos)

Cartilage supernatant fractions or QconCAT were detergent treated with 1% (*w*/*v*) Rapigest (Waters, Manchester, UK) for 10 min at 80 °C in 25 mM ammonium bicarbonate. In-solution tryptic digestion of protein samples was carried out following sequential reduction and alkylation in 3 mM DTT (60 °C for 10 min) and then 9 mM iodoacetamide (30 min in the dark at room temperature) with trypsin at a ratio of 1:50 protein: trypsin ratio overnight at 37 °C. Detergent inactivation was then assumed by incubating for 45 min at 37 °C with trifluoroacetic acid (VWR International, Lutterworth, Leicestershire, UK) to a final concentration of 0.5% (*v*/*v*). Following centrifugation (10 min, 15,000 × *g*) the soluble phase was retrieved and used for LC-MS/MS.

LC-MS/MS analysis was performed using nanoAcquity™ ultraperformance LC (Waters, Manchester, UK) on line to an LTQ-Orbitrap Velos (Thermo-Fisher Scientific, Hemel Hempstead, UK) via a ESI ion source containing a 10 μm coated Pico-tip emitter (Presearch LTD, Basingstoke, UK). Aliquots of tryptic peptides equivalent to 250 ng of cartilage secretome protein or 100 fmol of QconCAT (for QconCAT verification and validation) were loaded onto a 180 μm × 20 mm C_18_ trap column (Waters, Manchester, UK) at 5 μL/min in 99% solvent A (water plus 0.1% FA) and 1% solvent B (acetonitrile plus 1% FA for 5 min and subsequently back-flushed onto a C_18_ pre-equilibrated analytical column (75 μm × 15 mm Waters, Manchester, UK) using a flow rate of 0.3 μL/min. Xcalibur 2.0 software (Thermo -Electron, Hemel Hempstead, UK) was used to operate the LTQ-Orbitrap Velos in data-dependant acquisition mode. The survey scan was acquired in the Orbitrap with a resolving power set to 30,000 (at 400 *m*/*z*). MS/MS spectra were concurrently acquired on the 20 most intense ions from the high resolution survey scan in the LTQ. Charge state filtering >1 was used, where unassigned precursor ions were not selected for fragmentation. Fragmentation parameters in the LTQ were: normalized collision energy; 30, activation; 0.250, activation time; 10 ms and minimum signal threshold 500 counts with isolation width 2 *m*/*z*.

### 4.7. Peptide Identification

Raw spectra were converted to mascot generated files (mgf) using Proteome Discoverer software (Thermo, Hemel Hempstead, UK). The resulting mgf files were searched against either the Human IPI database, taxonomy; mammalian or QconCAT sequence databases using an in-house Mascot [12] server (Matrix Science, London, UK). Search parameters used were: peptide mass tolerances, 10 ppm; fragment mass tolerance, 0.6 Da; 1+, 2+ and 3+ ions; missed cleavages, 1; instrument type, ESI-TRAP. Modifications included were: fixed carbamidomethyl cysteine and variable; oxidation of methionine.

### 4.8. Selected Reaction Monitoring Optimization

The selected reaction monitoring (SRM) assay conditions were optimised using a XEVO TQ (Waters, Manchester, UK). Trypsin digestion of QconCAT was carried out as described previously [27]. The peptides were diluted with 97:3:0.1 ACN: water: FA and 100 fmol of peptides were loaded on the column. Optimisation was performed on a XEVO TQ operated with MassLynx 2.4 (Waters, Manchester, UK) coupled to a nanoAcquity™ UPLC (Waters, Manchester, UK). Peptides were loaded using partial loop injection for three minutes at a flow rate of 5 μL/min with 0.1% (*v*/*v*) formic acid onto a trapping column (Waters, C_18_, 180 μm × 20 mm). Samples were separated by a 30 min gradient of 97% A (0.1% (*v*/*v*) formic acid), 3% B (99.9% acetonitrile 0.1% (*v*/*v*) formic acid) to 60% A 40% B at a flow rate of 300 nL/min on a C_18_ analytical column (Waters, nanoACQUITY UPLC™ BEH C_18_ 75 μm × 150 mm 1.7 μm column). All transitions were acquired with the following parameters: 3 kV ion spray voltage; an 80 °C interface heater temperature; Q1 and Q3 operating at unit resolution. Cone voltages and collision energies were optimised for each peptide. Dwell times for transitions were determined automatically based on the number of co-eluting peptides but a minimum dwell time of 50 ms was maintained.

### 4.9. Protein Digestion and Quantification

For each sample 10 μg protein was detergent treated, reduced, alkylated and trypsin digested as described above. For SRM experiments, previously trypsin digested QconCAT was spiked into the samples. SRM experiments were conducted with 500 ng of tryptic analyte peptides spiked in with either 10 fmol, 1 fmol or 0.1 fmol heavy QconCAT, loaded onto column. MS analysis was commenced using the methods, parameters and gradients as described above. The ranging ensured that analyte:signal to noise were between 1:10 and 10:1 ratio of a QconCAT loading. MassLynx 2.4 (Waters, Manchester, UK) software was used to produce extracted ion chromatograms of the peptide transitions in order to compare the ratio of analyte to standard. Ratios were converted to fmol and then normalised to dry weight of cartilage explant.

### 4.10. Label-Free Peptide Quantification

The Thermo raw files of the acquired spectra were analysed by the Progenesis™ LC-MS software (version 4, Nonlinear Dynamics, Newcastle, UK) for label-free quantification. Progenesis™ LC-MS takes profile data of the MS scans and transforms them to peak lists. One sample was selected as a reference after checking the 2-D mapping (*m*/*z versus* retention time), and the retention times of the other samples within the experiment were aligned. This was undertaken by studying the chromatogram and aligning on the major peaks. Features without the 1+, 2+, 3+ and 4+ charge and isotope peaks of ≤2 were masked and excluded from further analysis. Samples were then divided into the appropriate groups using between subject design. Raw abundances of all features were normalised which corrects for factors due to experimental variation.

Following feature picking, we picked the top three spectra for each feature. These were exported from Progenesis™-LC-MS and utilized for peptide identification with a locally implemented Mascot server (version 2.3.01, University of Liverpool, Liverpool, UK, 2010) in the Unihuman database. Search parameters used were: 10 ppm peptide mass tolerance and 0.6 Da fragment mass tolerance; one missed cleavage allowed; fixed modification; carbamidomethylation; variable modifications; methionine oxidation. Mascot determined peptides with ion scores of 33 and above and only proteins with at least one unique peptide ranked as a top candidate were considered and re-imported into Progenesis™ software. Following the import of the Mascot results for quantification, statistical analysis was performed on all detected features using transformed normalized abundances for one-way analysis of variance (ANOVA). The total cumulative abundance was calculated by summing the abundances of all peptides allocated to the respective protein. All peptides (with Mascot score > 33 and *p <* 0.05) of an identified protein were included and the protein *p* value (one-way ANOVA) was then performed on the sum of the normalized abundances for all runs. ANOVA values of *p <* 0.05 and, additionally, regulation of >2-fold or <0.5-fold were regarded as significant.

### 4.11. Gene Ontology

Using DAVID gene ontology (GO) analysis, all genes identified were loaded into the functional annotation chart.

### 4.12. Western Blot Analysis Validation

Western blotting with matrix metalloproteinase 3 (MMP-3) was used as a complementary methodology to validate our results. Volumes of cartilage explant supernatant from three donors and conditions were adjusted to represent equal dry weights of cartilage. Human recombinant MMP-3 (Merckt, Darmstadt, Germany) was used as a positive control. Samples were heated to 80 °C for 10 min in NuPAGE^®^ LDS sample buffer (Invitrogen, Paisley, UK) and electrophoresed for 1 h at 200 V under reducing conditions on a Novex 4%–12% SDS-PAGE gel (Invitrogen, Paisley, UK). Protein transfer to nitrocellulose was performed using the Invitrogen X Cell Sure Lock apparatus according to standard protocol. Membranes were blocked with TBS (pH 7.4) containing 0.1% Tween-20 (Invitrogen, Paisley, UK) (TBST) and 5% dried skimmed milk for 1 h at room temperature. A goat polyclonal MMP-3 primary antibody (Abcam, Cambridge, UK) was diluted to 1:1000 in milk powder/Tween and added to the membrane for overnight incubation at 4 °C. Following washing in TBST, membranes were incubated for 1 h at room temperature with the secondary antibody conjugated to horseradish peroxidise (HRP); polyclonal rabbit anti-goat IgG HRP (Abcam, Cambridge, UK) at 1:5000 diluted with TBST containing 5% dried skimmed milk. Chemiluminescence was used to detect the protein bands using Western Lightning™ and Western Lightning_Plus Chemiluminescence reagents (Perkin Elmer, Beaconsfield, IA, USA). ImageJ software (http://rsbweb.nih.gov/ij/) was used to quantify bands using densitometry. The relative intensity of IL-1β treated samples were compared to the control for each donor.

### 4.13. Statistical Analysis

Statistical analysis for absolutely quantified peptides was undertaken using mixed effects linear regression to allow for donors with significant biological variation with SPLUS 6.1 software (NCSS Software, Kaysvill, UT, USA, 2001). Statistical testing for quantitative western blotting was undertaken following normality testing with a paired Student’s *T* test using Minitab 15 (Minitab Software, Coventry, UK, 2001) and Excel software (Microsoft, Redmond, WA, USA, 2009).

## 5. Conclusions

This study is the first to combine relative and absolute protein quantification in the analysis of the human OA secretome. It enabled the identification of a cohort of proteins expressed by OA cartilage with possible roles in its pathogenesis. A human cartilage QconCAT was designed, expressed and validated, which enabled the absolute levels of important proteins in the study of OA to be quantified. The QconCAT provides a tool for the precise definition of some matrix proteins and proteases important in the pathogenesis of OA.

## Figures and Tables

**Table 1:** A number of differentially expressed proteins were identified by Progenesis™ LC-MS software. Proteins shown were identified with ≥2 unique peptides and with a >2-fold change in normalised abundance.

Highest Mean Condition	Accession	Description	Max Fold Change	ANOVA (*p*)
**Treatment**	P09341	Growth-regulated alpha protein	58.99	0.01
	P08254	Stromelysin-1	5.70	0.02
	Q61PR1	LYR motif-containing protein 5	3.89	0.03
	P01876	Ig alpha-1 chain C region	3.06	0.00

**Control**	P36222	Chitinase-3-like protein 1	9.64	0.04
	P08571	Monocyte differentiation antigen CD14	4.53	0.01
	Q14UF6	Decay-accelerating factor splicing variant 1	4.11	0.02
	Q5H9A7	TIMP metalloproteinase inhibitor 1	3.09	0.03
	P01034	Cystatin-*C*	2.40	0.05

**Table 2:** Represents peptide types as determined by SRM experiments. Q-peptides are classified for quantification purposes as A, B, C. “Type A” native and QconCAT peptides are detected. “Type B” are peptides detected for the QconCAT but not in native form and when neither QconCAT nor native peptides are detected a “Type C” classification is given (ADAMTS; A disintegrin and metalloproteinase with thrombospondin motifs).

Protein	Q-peptide amino acid sequence	Peptide classification
Aggrecan	EVVLLVATEGR	A
Cartilage oligomeric matrix protein	DTDLDGFPDEK	A
Cartilage oligomeric matrix protein	SSTGPGEQLR	A
Fibromodulin	IPPVNTNLENLYLQGNR	A
Matrix metalloproteinase-1	SQNPVQPIGPQTPK	A
Matrix metalloproteinase-3	IVNYTPDLPK	A
Metalloproteinase inhibitor 1	GFQALGDAADIR	A
Plasminogen	EAQLPVIENK	A
ADAMTS1	DAEHYDTAILFTR	B
ADAMTS1	GPEVTSNAALTLR	B
ADAMTS4	FVETLVVADDK	B
ADAMTS4	NPVSLVVTR	B
ADAMTS5	LPLAAVGPAATPAQDK	B
ADAMTS5	GLVQNIDQLYSGGGK	B
Aggrecan	LEGEVFFATR	B
Cathepsin D	LVDQNIFSFYLSR	B
Cathepsin D	YSQAVPAVTEGPIPEVLK	B
Cathepsin K	SNDTLYIPEWEGR	B
Link protein	GGSDSDASLVITDLTLEDYGR	B
Metalloproteinase inhibitor 1	FVGTPEVNQTTLYQR	B
Metalloproteinase inhibitor 3	WDQLTLSQR	B
Metalloproteinase inhibitor 4	GHLPLR	B
Cathepsin K	VGPVSVAIDASLTSFQFYSK	C
Fibromodulin	LYLDHNNLTR	C
Link Protein	FYYLIHPTK	C
Matrix metalloproteinase-1	DGFFYFFHGTR	C
Matrix metalloproteinase-13	LHPQQVDAELFLTK	C
Metalloproteinase inhibitor 3	YQYLLTGR	C
Metalloproteinase inhibitor 4	LEANSQK	C
Plasminogen	HSIFTPETNPR	C

**Table 3:** Human cartilage QconCAT signature peptides in QconCat context order. The three amino acids found adjacent to the *N* and *C* termini of the Q-peptide within the native protein are indicated (Matrix metalloproteinase (MMP), collagen (Col), a disintegrin and metalloproteinase with thrombospondin motifs (ADAMTS), tissue inhibitor of metalloproteinase (TIMP)).

Peptide order	Protein	Protein Accession	Q-peptide amino acid sequence
1	MMP16	ENSP00000286611	GIPESPQGAFVHK
2	MMP16	ENSP00000286611	EGHSPPDDVDIVIK
3	CathepsinD	ENSP00000236671	LVDQNIFSFYLSR
4	COMP	ENSP00000222271	DTDLDGFPDEK
5	Col11a2	ENSP00000372565	LGVPGLPGYPGR
6	Fibromodulin	ENSP00000347041	IPPVNTNLEN LYLQGNR
7	MMP3	ENSP00000299855	IVNYTPDLPK
8	ADAMTS1	ENSP00000284984	DAEHYDTAILFTR
9	ADAMTS4	ENSP00000356975	FVETLVVADDK
10	CathepsinD	ENSP00000236671	YSQAVPAVTEGPIPEVLK
11	Link protein	ENSP00000274341	GGSDSDASLVITDLTLEDYGR
12	MMP3	ENSP00000299855	YLENYYDLK
13	ADAMTS5	ENSP00000284987	GLVQNIDQLYSGGGK
14	TIMP3	ENSP00000266085	WDQLTLSQR
15	TIMP4	ENSP00000287814	GHLPLR
16	CathepsinK	ENSP00000271651	SNDTLYIPEWEGR
17	Link Protein	ENSP00000274341	FYYLIHPTK
18	COMP	ENSP00000222271	SSTGPGEQLR
19	Plasminogen	ENSP00000308938	HSIFTPETNPR
20	MMP13	ENSP00000260302	LHPQQVDAELFLTK
21	MMP13	ENSP00000260302	SYYHPTNLAGILK
22	Plasminogen	ENSP00000308938	EAQLPVIENK
23	Col9a1	ENSP00000349790	VVGSATLQVAYK
24	TIMP4	ENSP00000287814	LEANSQK
25	MMP1	ENSP00000322788	DGFFYFFHGTR
26	ADAMTS1	ENSP00000284984	GPEVTSNAALTLR
27	ADAMTS4	ENSP00000356975	NPVSLVVTR
28	Aggrecan	ENSP00000268134	LEGEVFFATR
29	Fibromodulin	ENSP00000347041	LYLDHNNLTR
30	TIMP1	ENSP00000218388	GFQALGDAADIR
31	TIMP1	ENSP00000218388	FVGTPEVNQTTLYQR
32	TIMP3	ENSP00000266085	YQYLLTGR
33	Col11a2	ENSP00000372565	VARPAQLSAPTR
34	ADAMTS5	ENSP00000284987	LPLAAVGPAATPAQDK
35	Aggrecan	ENSP00000268134	EVVLLVATEGR
36	CathepsinK	ENSP00000271651	VGPVSVAIDASLTSFQFYSK
37	Col2a1	ENSP00000338213	GAQGPPGATGFPGAAGR
38	Col2a1	ENSP00000338213	GPPGPQGAR
39	Col9a1	ENSP00000349790	GVQGEQGATGLPGVQGPPGR
40	MMP1	ENSP00000322788	SQNPVQPIGPQTPK
